# Retraction Note: Circular RNA circDLG1 contributes to HCC progression by regulating the miR-141-3p/WTAP axis

**DOI:** 10.1007/s10142-024-01375-2

**Published:** 2024-05-10

**Authors:** Qian Wang, Wei Yu, Tao Wang, Changshan Huang

**Affiliations:** Department of Hepatobiliary and Pancreatic Surgery, The Affiliated Cancer Hospital of Zhengzhou University, Zhengzhou, 450008 China

**Retraction Note: Functional & Integrative Genomics (2023) 23:179**

10.1007/s10142-023-01096-y

The Editor-in-Chief and the publisher have retracted this article. The article was submitted to be part of a guest-edited issue. An investigation by the publisher found a number of articles, including this one, with a number of concerns, including but not limited to compromised editorial handling and peer review process, inappropriate or irrelevant references or not being in scope of the journal or guest-edited issue. Based on the investigation’s findings, the Editor-in-Chief therefore no longer has confidence in the results and conclusions of this article.

The author, Qian Wang, has stated that all authors disagree with this retraction.

